# The Spectrum of Polyoma Virus and Polyoma Virus‐Like Changes in Urine Cytology: When Should an Atypical Diagnosis Be Considered

**DOI:** 10.1002/dc.25494

**Published:** 2025-06-09

**Authors:** Yaogong Li, Yonca Kanber, Derin Caglar, Wassim Kassouf, Manon Auger, Fadi Brimo

**Affiliations:** ^1^ University Hospital of Northern British Columbia Prince George British Columbia Canada; ^2^ Department of Pathology McGill University and McGill University Health Center Montreal Quebec Canada; ^3^ Department of Surgery (Urology) McGill University Health Center Montreal Quebec Canada

**Keywords:** high‐grade urothelial carcinoma, Paris system, polyoma virus, urine cytology

## Abstract

**Background:**

While the Paris system for reporting urinary cytology states that cells with well‐recognized and typical changes of polyoma virus (PV) infection should not lead to a diagnosis of ‘atypia, the prognostic significance of PV‐like features is unknown.

**Methods:**

Included were 284 urine cytology cases with PV or PV‐like changes. Four cell types were identified. Cell A was typical of PV infection, cell B had a spider‐web chromatin pattern, and cells C and D were degenerated cells with clumpy chromatin or homogeneous severely hyperchromatic nuclei, respectively. Events were correlated with a subsequent histological diagnosis of high‐grade urothelial carcinoma (HGUC).

**Results:**

47% of cases had more than one cell type. The most common cell was D (64%) and the least common was A (27%). The most common cell to be present in isolation was D (*n* = 109, 60%). 92% of cases with cell A had other cell types. Overall, the presence of cell A was associated with a benign follow‐up (1.2% association with HGUC). There was a gradual increase in the association with a HGUC diagnosis in cases with cells B (PPV = 8%), C (PPV = 15.5%), and D (PPV = 44%). The presence of only cells B, C, or D was predictive of HGUC in 22.4%, 33.3%, and 61.5%, respectively.

**Conclusions:**

Cases with cell type A can be confidently diagnosed as ‘negative for HGUC’, even in the presence of associated cells with PV‐like changes. Cases with cells displaying PV‐like features (cell type B, C, or D) in the absence of type A features may represent degenerated HGUC cells.

## 1 Background

Urothelial carcinoma (UC) is among the top ten most common cancer types globally, with approximately half a million new cases diagnosed annually [1]. The majority of UC diagnoses (90%) are made in those 55 years of age and older, and it is four times more common in men than in women. In addition to cystoscopy and upper tract imaging, urine cytology has been an integral part of the initial workup for patients with hematuria or for routine surveillance for patients with a history of UC [2, 3].

The Paris System for Reporting Urinary Cytology (TPS), first published in 2016 [4], developed a standardized approach to the interpretation of urinary cytology samples with the goal of detecting high‐grade urothelial carcinoma (HGUC). According to TPS, specimens showing cells with typical changes of polyomavirus cytopathic effect (PV‐CPE) do not need an ‘atypical’ label and can be considered ‘negative for HGUC’, despite some overlapping morphological features with HGUC cells (5). According to TPS, polyomavirus infected cells are usually single and enlarged, with a homogeneous basophilic inclusion occupying most of the nuclear area. Nuclear membranes are smooth and regular, and may sometimes appear thickened due to chromatin margination. When these cells degenerate, the basophilia appears as the chromatin extrudes, leaving a framework or ‘spider web’ [4, 5].

Although this description seems straightforward, its integration to routine practice has not proven to be always easily applicable in our center. With most of the urine specimens received being voided urines, the number of specimens showing cellular degeneration with some features suggestive of PV‐CPE is not negligible. In fact, whether to consider cases with single hyperchromatic degenerated cells with spider‐web chromatin pattern as ‘negative’ and falling within the spectrum of PV‐infection or ‘atypical’ is a frequent cause of intra‐ and inter‐departmental consultation, which is usually solved by consensus opinion, rather than by an evidence‐based and data‐driven approach.

To date, only two studies [6, 7] have addressed the significance of PV‐like changes in urine cytology with somewhat differing results. However, none has specifically detailed the morphological features that fall within the spectrum of definitive PV‐infection‐related changes. With the aim of solving this issue, we conducted a morphological analysis of a large number of urine cytology cases with cells either typical or reminiscent of PV‐CPE, and we correlated the findings with subsequent clinical or histological follow‐up, with HGUC considered as a positive outcome.

## 2 Methods

### 2.1 Patients Recruitment

We conducted a search of all the urine cytology specimens diagnosed as ‘atypical urothelial cells’ in the years 2018–2019 using the McGill University Health Center laboratory electronic system. Only cases with subsequent biopsy follow‐up within one year of the cytological diagnosis were included. Of those, after cytological review, the only included cases were those that had single cells reminiscent of PV‐CPE as detailed below. Cases in which the nature of atypia warranted a ‘suspicious for HGUC’ or a ‘positive for HGUC’ were excluded as the question of whether to diagnose the case as ‘benign’ or ‘atypical’ would not be applicable. Also excluded were cases with cells falling within the ‘atypical urothelial cell’ TPS category in which the atypical cytomorphological changes did not overlap with PV‐CPE as those cases were irrelevant for the purpose of this specific study. To increase the study cohort, we also retrieved all cases in which the word ‘polyomavirus’ appeared between the years 2012–2022. Considering that the rate of subsequent biopsies in this group was null, we also included clinical follow‐up as an outcome in this subset of cases. A negative clinical follow‐up was defined as no evidence of HGUC clinically within at least one year from the cytology specimen, provided the patient had negative cystoscopy and no evidence of hematuria.

Age, type of specimen (voided versus instrumental/cystoscopic) and clinical history (hematuria, transplantation, surveillance or others) were recorded for each cytology specimen. The surveillance cohort included patients who had a previous diagnosis of urothelial carcinoma and who were followed by repeat cytology and cystoscopy. The non‐surveillance cohort included patients with no known history of urothelial carcinoma, such as patients presenting with hematuria, renal stones, urinary tract infections, and post‐renal transplantation. In the cases with histological follow‐ups, carcinoma in situ, non‐invasive high‐grade papillary urothelial carcinoma, invasive high‐grade papillary urothelial carcinoma, were defined as ‘high‐grade urothelial carcinoma’; whereas low grade papillary urothelial carcinoma, reactive changes and cases with atypical changes were defined as ‘benign/low grade’.

### 2.2 Criteria for Cell Type Identification

**FIGURE 1:**
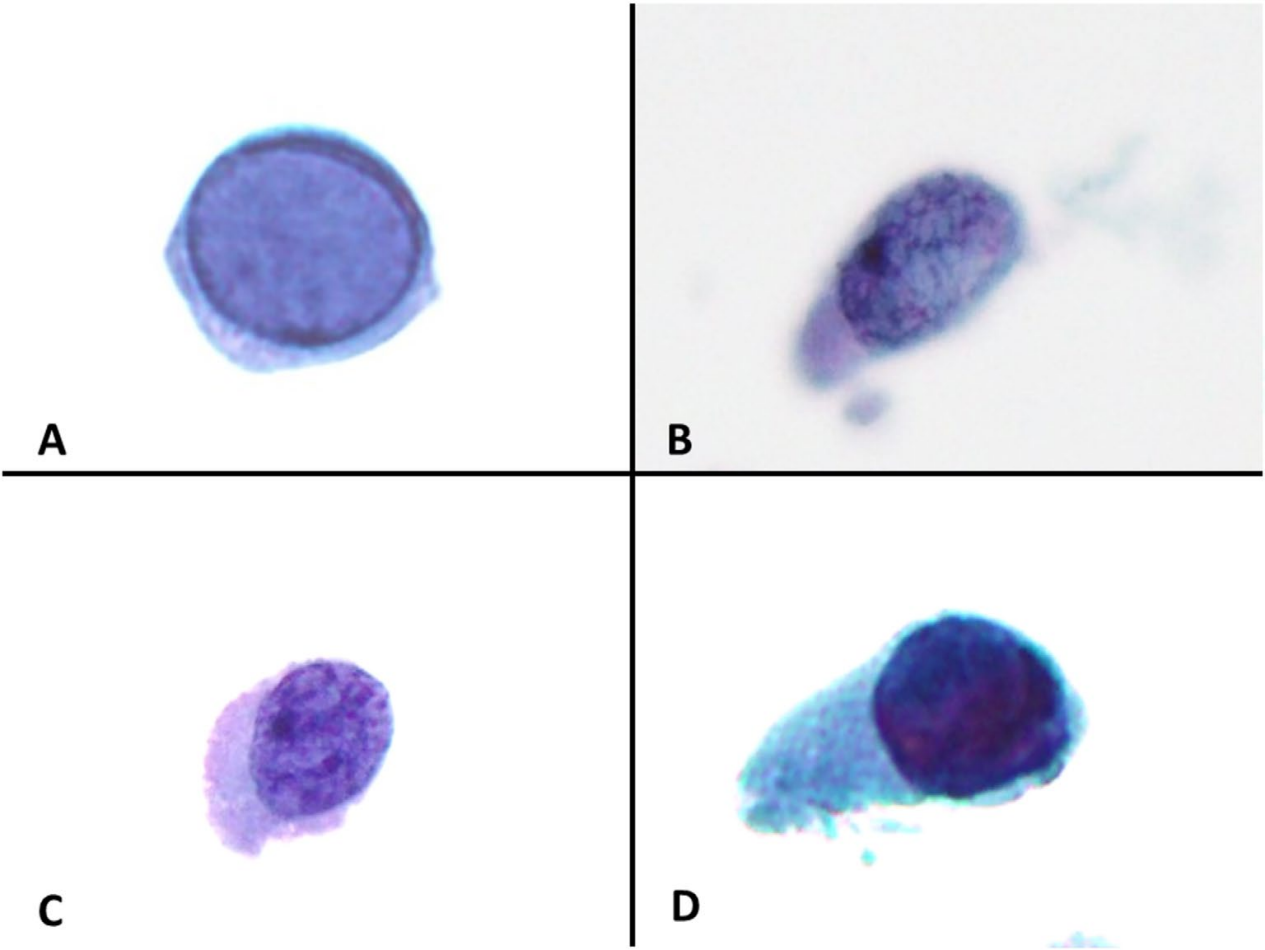
A. *Type A cell* is a single cell with changes typical of polyoma infection: well preserved cells with smudgy homogeneous basophilic nuclear inclusion, marginated chromatin, and smooth nuclear membranes. B. *Type B cell* is a degenerated cell with normo‐ to hyperchromatic nuclei, evaluable chromatin details, and filamentous spider‐web chromatin pattern. C. *Type C cell* is a degenerated cell with normochromatic to hyperchromatic nuclei, evaluable chromatin details, and granular to clumpy chromatin. D. *Type D cell* is a degenerated cell with homogeneous severely hyperchromatic nuclei, in which the chromatin pattern or distribution could not be evaluated. [Color figure can be viewed at wileyonlinelibrary.com]

All slides were reviewed by two cytopathologists (YL, FB). All the cells of interest were single cells with a high nuclear‐to‐cytoplasmic ratio (above 0.5). Using strict criteria, the cells were categorized into four types as follows: type A (Figure 1A) consisted of single cells showing changes typical of PV‐CPE: well‐preserved cells with smudgy homogeneous basophilic nuclear inclusion, marginated chromatin, and smooth nuclear membranes. Type B (Figure 1B) consisted of degenerated cells with normo‐to hyperchromatic nuclei, evaluable chromatin details, and filamentous spider‐web chromatin pattern. Type C (Figure 1C) consisted of degenerated cells with normochromatic to hyperchromatic nuclei, evaluable chromatin details, and granular to clumpy chromatin. Type D (Figure 1D) consisted of degenerated cells with homogeneous severely hyperchromatic nuclei, in which the chromatin pattern or distribution could not be evaluated.

### 2.3 Statistical Analysis

Different cell types and cell types' combinations were correlated with a subsequent ‘high‐grade urothelial carcinoma’ diagnosis. For each category, the ratio of patients who had subsequent ‘high‐grade urothelial carcinoma’ divided by the total case number in that category was calculated as ‘positive predictive value/PPV’. Different PPVs of different categories were compared using two‐variable Chi‐Square test (X2), using the online JMP software and a *p* value ≤ 0.05 as statistically significant. Institutional ethics approval was obtained (2017–2612).

## 3 Results

### 3.1 Cohort Characteristics

In total, 1856 specimens from 1330 patients were initially included. Of those, 358 cases had an appropriate histological or clinical follow‐up and were reviewed. Of those, 17 cases were reclassified to another TPS category; 10 from AUC to NHGUC, and 7 cases from AUC to SHGUC. 57 cases had atypical cytological changes that did not overlap with PV‐CPE and were also excluded. The final cohort consisted of 284 urine cytology cases belonging to 228 patients, with PV‐CPE or PV‐CPE‐like changes. 176 patients were males and 52 females. Average age was 67.8 years (range = 10–94). Average age for any case with type A cell was 61.6 years, in comparison to 67.2, 67.7, and 68 for cases with any cell B, C, and D, respectively. The clinical context was surveillance following a diagnosis of urothelial carcinoma in 163 patients, hematuria/urinary symptoms in 70 patients, and post‐renal transplantation in 51 patients. 239 specimens were voided (84%), and 45 were instrumented (18%). The follow‐up was histological in 233 patients and clinical in 51 patients (only in post‐renal transplantation patients).

### 3.2 Cytological Features and Cell Type

The most common cell identified in the cohort was type D (*n* = 181, 64%) followed by type C (*n* = 118, 42%), B (*n* = 109, 38%), and A (*n* = 78, 27%). 47% of cases had more than one cell type within the same specimen. The most common combination of cells within individual specimens was B + C (*n* = 20), followed by B + C + D (*N* = 18), and C + D (*n* = 17). The least common combination within individual specimens was A + D (*n* = 4). The most common cell to be present in isolation was D, which was the only ‘abnormal’ cell‐type present in 109 specimens (38% of the entire cohort). In contrast, 92% of urine specimens with cell A had other cell types, the most common being B, which was present in 60% (*n* = 47) of cases, followed by C (*n* = 45, 58%) and D (*n* = 31, 40%). In summary, while it was common for cell type D to be seen in isolation, the opposite was true for cell type A.

In the post‐renal transplantation group (*n* = 51), the most common cell identified was A (*n* = 38, 73%). Of those, 95% of cases had other cell types. The most common cell associated with A in this context was C (*n* = 25, 66%), followed by B (*n* = 22, 58%), and D (*n* = 17, 45%). 14 cases post‐transplant did not have cell A. Of those, the most common cell present was C (*n* = 11, 79%), followed by D (*n* = 10, 71%), and B (*n* = 7, 50%).

### 3.3 Follow‐Up (Table 1)

Overall, the presence of cell A was almost invariably associated with a benign follow‐up (positive predictive value for HGUC or PPV = 1.2%). There was a gradual increase in the association with a HGUC diagnosis in cases with cells B (PPV = 8%), C (PPV = 15.5%), and D (PPV = 44%). This difference was significant when comparing A with B (*p* = 0.003), A with C (*p* = 0.001), A with D (*p* = 0.0001), B with D (*p* = 0.0001), and C with D (*p* = 0.0001). B and C cells had comparable rates of subsequent HGUC diagnosis (8% vs. 15.5%, *p* = 0.1). Interestingly, the mere presence of cells A in any case independent of the presence/absence of other cell types resulted in a statistically significant decrease in the association with a HGUC outcome. As an example, for cases with cell B type, the association with HGUC diagnosis was 14.5% in the absence of A cells and 0% in its presence (*p* = 0.02). Cases with only‐type B cells had a 22.3% risk of a HGUC follow‐up. Similarly, the presence of cell D in association with A was associated with subsequent HGUC in only 3.5% of cases, while the association was 52% in the absence of cell A. This finding indirectly indicates that cell A morphology likely represents genuine PV infection, while other PV‐CPE‐like changes, including cells with spider web chromatin pattern (cell B) may represent either PV‐CPE or degenerated neoplastic cells.

Interestingly, by dividing the cohort into patients on surveillance following a diagnosis of HGUC versus patients with no history of HGUC (excluding post‐transplants), the surveillance cohort showed similar findings to those described above. However, in the non‐surveillance cohort, the association with HGUC was comparable between cases with cells A (PPV = 0%), B (PPV = 3%), and C (PPV = 7%), even in the absence of cell A for the latter two groups. In contrast, cell D maintained its significant association with HGUC (PPV = 51%) in the absence of cell A.

In the post‐transplantation population (*n* = 51), all patients had a benign follow‐up.

## 4 Discussion

The TPS outlines the characteristic features of PV‐infection, which are generally straightforward and reproducible. It also states that PV‐infected cells may have a spider‐web chromatin pattern, and that cells with PV‐CPE should be classified as ‘negative for HGUC’ [4, 5]. However, what is not addressed in the literature is whether cells with spider‐web chromatin pattern or degenerated cells that are reminiscent of PV‐CPE, without entirely fulfilling the described criteria of PV‐CPE can be confidently diagnosed as ‘negative for HGUC’, or whether an ‘atypical’ diagnosis should be considered. In addition, whether the clinical context related to the possibility of PV‐infection can be incorporated in this decision‐making process remains an unanswered question. What is also unclear is how to deal with cases showing cells with typical PV‐CPE in addition to degenerated hyperchromatic cells that would otherwise be called ‘atypical’, according to the criteria detailed in the AUC category chapter [5]. In our group's experience, the question of whether to classify such cases into the AUC category or to the ‘negative for HGUC, PV‐CPE’ category is not uncommonly raised. This is particularly relevant in our cytopathology practice setting in which most of the urine cytology specimens received are voided urines, a significant proportion of which have degenerated cells.

With those questions in mind, we studied a cohort of patients presenting with such single cells in their urine cytology specimens, and we correlated the findings with clinical outcome, making this study the first to thoroughly evaluate and report the spectrum of cellular changes that raise the possibility of PV‐infection in urine cytology.

The first observation made from our results was the frequent presence of several cell types (A to D) within individual urine cytology specimens, with half of the cohort having more than one cell type. The most common cell type to be associated with others was type A (92% association), followed by C (85%), B (83%), and D (40%). This illustrates the diagnostic complexity of those cases in which cytopathologists are confronted with a wide morphological nuclear and cellular spectrum in specimens that are often degenerated and poorly preserved.

The second important observation was the strong association of cell type A with a benign diagnosis in the cohort, independent from the clinical context, and independent from the presence or absence of other cell types. In comparison, our data show that other cell types, including those with spider‐web chromatin pattern cannot be reliably diagnosed as negative for HGUC. Indeed, of the 17 cases with cells B‐only, 22.3% had subsequent HGUC, indicating that they may represent degenerated malignant urothelial cells. Although we did not correlate the cytological findings with the serological results of PV‐infection (which were not routinely performed and rarely available), those findings highlight that the only reliable cellular changes that can be confidently diagnosed as ‘negative for HGUC’ and that are likely to be consistent with PV‐CPE are those with cell A features.

The third interesting finding was the noted gradual increase in the association with HGUC on follow‐up in cases with cells B, C, and D, when encountered in the absence of type A cells. Those findings were also true for cases with cells B‐only (PPV = 22.3%), C‐only (PPV = 33.3%), and D‐only (PPV = 61.5%). Despite this, the mere presence of cell A in any case resulted in a drastic switch to a benign outcome, including those specimens that had cell D morphology. This suggests that in the presence of typical PV‐CPE, one has to accept a certain degree of atypia in the background cells, and that even in the presence of what otherwise would be considered an ‘atypical urothelial cell’, such cases are better classified as ‘negative for HGUC’. Also, our findings emphasize the limitation of rendering definitive diagnoses based on individual cells' cytological features, without taking into account the overall spectrum of cellular changes noted in the specimen, specifically in the context of voided urine cytology specimens with PV‐CPE or PV‐CPE‐like features.

The fourth observation pertained to the specimens obtained in post‐transplantation patients. Not only did all those patients have a benign outcome, independent of the cell type present; what was also interesting was the absence of cell A type in 14% of such cases. This was the first indication in the study that the clinical history needed to be incorporated in the interpretation of those specimens. This was also confirmed by obtaining differing results in the surveillance versus non‐surveillance cohorts. In patients who did not have a previous history of HGUC (hematuria, urinary tract infection, urinary stones), the association of cells A, B, and C with HGUC was comparable (0%, 3%, and 7%, respectively), in contrast to cell D, which was 45% predictive of a HGUC outcome. In comparison, in patients with a previous history of HGUC, cases with cells B, C, and D had a significantly higher association with HGUC than those with cell A (PPV of 4% for cell A, 18% for cell B, 31% for cell C, and 54% for cell D).

Only two large previous studies have systematically addressed the issue of diagnosing and reporting PV‐CPE and the association with HGUC [6, 7]. In the *Allison* et al. [6] paper, cases with PV‐CPE changes have traditionally been included in the AUC category prior to the study. Two cytopathologists conducted a cytological review and reclassified 107 AUC cases that had a clinical follow‐up, into benign (62.6% of cases) for those showing PV‐CPE, and AUC (37.4% of cases) when other atypical features were present. The rate of subsequent HGUC was 6% for the cases reclassified as benign and 10% for the AUC category, a difference which was not statistically significant. Interestingly, when a non‐surveillance subset of patients with no prior history of UC was analyzed, cases with PV‐CPE had a 7% association with subsequent HGUC, in comparison to 3% only for the AUC category, rates that were comparable to those of a second AUC institutional control group (7.3%). The study concluded that a small number of patients with PV‐CPE may be classified as benign, but advocated against such reclassification in non‐surveillance patients due to the increased risk of HGUC. It also called for a better refinement of the cytological criteria to what constitutes genuine PV infection. Those findings, which conflicts with ours, are likely explained by the different methodologies and designs of both studies. Importantly, no clear cytological description of what falls within the spectrum of PV‐CPE was given in *Allison,* et al. study [6]. During the review, 38.3% of cases needed jurisdiction by a third cytopathologist before deciding whether to label the case as ‘negative’ or ‘AUC’. This is likely a reflection of the lack of well‐established criteria to what was to be considered PV‐related features or not. Also, it is unclear how the authors dealt with cases showing cells with varying degrees of nuclear and chromatin changes, which are a frequent finding, as highlighted in the current series. Lastly, the correlation was made with a follow‐up biopsy up to five years following the initial diagnosis, in comparison to a maximum of one year in the current study. We have chosen to restrict the histological follow‐up to one year, in order to minimize contaminating the results by newly growing high‐grade tumors after one‐year follow‐up, that would be unrelated to the included cytology cases. The second study is that of *Lu,* et al. [7] in which the authors compared the outcome of four cytological categories: NHGUC (*n* = 252), NHGUC with PV‐CPE (*n* = 234), AUC (*n* = 255), AUC with PV‐CPE (*n* = 64). The overall risk of subsequent HGUC was 6%, 6.8%, 23.5%, and 12.5%, respectively, indicating that the presence of PV‐CPE does not increase the risk of HGUC. This study supported the recommendations of TPS in considering PV‐CPE cases as negative, and is comparable to the findings of the current study. Similar to *Allison,* et al. the cytological criteria were not detailed, and it is unclear whether cases with cells showing spider‐web chromatin (type B) were labelled as NHGUG or AUC. The follow‐up which included clinical, cytological or histological follow‐up over 5 years post‐cytology is also significantly different from the criteria in the current study.

The main aim of the current study was PV‐infection‐related changes. Consequently, we excluded cases with atypical cytological features that were not reminiscent of PV‐infection. Therefore, the predictive rates of different categories with HGUC (shown in Tables 1, 2, 3) are not reflective of all urine cytology cases with a NHGUC or AUC diagnoses seen in our laboratory. In addition, this study also excluded cases with non‐degenerated atypical cells that fulfilled the criteria of ‘suspicious’ or ‘positive for HGUC’. Therefore, our findings are only applicable to cases that do not qualify as ‘suspicious or positive for HGUC’. This is particularly relevant; as anecdotal cases of PV‐associated UCs have been described in the literature [8, 9]. As such, the presence of type A‐cells should theoretically not be considered as incompatible with a diagnosis of HGUC, although such association remains extremely rare in routine practice.

**TABLE 1:** Different cell types combination in association with outcome for the entire cohort.

Entire Cohort (*n* = 284 urine specimens)		
	F/U: High‐grade N (PPV)	F/U: benign/LG N (PPV)
Any case with type A (*N* = 78)	**1 (1.2%)**	**77 (98.8%)**
Any case with type B (*n* = 109)	**9 (8%)**	**100 (92%)**
B in the presence of A (*n* = 47)	0 (0%)	47 (100%)
B in the absence of A (*n* = 62)	9 (14.5%)	53 (85.5%)
B only (*n* = 18)	4 (22.3%)	14 (77.7%)
Any case with type C (*n* = 118)	**18 (15.5%)**	**100 (84.5%)**
C in the presence of A (*n* = 45)	1 (2.5%)	44 (97.5%)
C in the absence of A (*n* = 73)	17 (24%)	56 (76%)
C only (*n* = 18)	6 (33.3%)	12 (66.7%)
Any case with type D (*n* = 181)	**79 (44%)**	**102 (56%)**
D in the presence of A (*n* = 31)	1 (3.5%)	30 (96.5%)
D in the absence of A (*n* = 150)	78 (52%)	72 (48%)
D only (*n* = 109)	67 (61.5%)	42 (38.5%)

Voided urine (*n* = 239 urine specimens)		
	F/U: High‐grade N (PPV)	F/U: benign/LG N (PPV)
Any case with type A (*N* = 72)	**1 (1.5%)**	**71 (98.5%)**
Any case with type B (*n* = 104)	**8 (7.7%)**	**96 (92.3%)**
B in the presence of A (*n* = 46)	0 (0%)	46 (100%)
B in the absence of A (*n* = 58)	8 (13.7%)	50 (86.3%)
B only (*n* = 17)	4 (23.5%)	13 (76.5%)
Any case with type C (*n* = 106)	**15 (14%)**	**91 (86%)**
C in the presence of A (*n* = 40)	1 (2.5%)	39 (97.5%)
C in the absence of A (*n* = 65)	14 (21.5%)	51 (78.5%)
C only (*n* = 17)	6 (35%)	11 (65%)
Any case with type D (*n* = 145)	**59 (40.6%)**	**86 (59.4%)**
D in the presence of A (*n* = 29)	1 (3.5%)	28 (96.5%)
D in the absence of A (*n* = 116)	58 (50%)	58 (50%)
D only (*n* = 80)	49 (61%)	31 (39%)

Instrumented urine (*n* = 45 urine specimens)		
	F/U: High‐grade N (PPV)	F/U: benign/LG N (PPV)
Any case with type A (*N* = 6)	**0 (0%)**	**6 (100%)**
Any case with type B (*n* = 5)	**1 (20%)**	**4 (80%)**
B in the presence of A (*n* = 1)	0 (0%)	1 (100%)
B in the absence of A (*n* = 4)	1 (25%)	3 (75%)
B only (*n* = 1)	0 (0%)	1 (100%)
Any case with type C (*n* = 12)	**3 (25%)**	**9 (75%)**
C in the presence of A (*n* = 5)	0 (0%)	5 (100%)
C in the absence of A (*n* = 8)	3 (37.5%)	5 (62.5%)
C only (*n* = 1)	0 (0%)	1 (100%)
Any case with type D (*n* = 36)	**20 (55.5%)**	**16 (45.5%)**
D in the presence of A (*n* = 2)	0 (0%)	2 (100%)
D in the absence of A (*n* = 34)	20 (58.8%)	14 (41.2%)
D only (*n* = 29)	18 (62%)	11 (38%)

Abbreviations: F/U: follow‐up; LG: Non‐invasive papillary low‐grade urothelial carcinoma; PPV: positive predictive value.

**TABLE 2:** Different cell types combination in association with outcome for the surveillance cohort.

Surveillance (*n* = 163 urine specimens)		
	F/U: High‐grade N (PPV)	F/U: benign/LG N (PPV)
Any case with type A (*n* = 25)	**1 (4%)**	**24 (96%)**
Any case with type B (*n* = 44)	**8 (18%)**	**36 (82%)**
B in the presence of A (*n* = 15)	0	15 (100%)
B in the absence of A (*n* = 29)	8 (28%)	21 (72%)
B only (*n* = 11)	4 (36%)	7 (64%)
Any case with type C (*n* = 52)	**16 (31%)**	**36 (69%)**
C in the presence of A (*n* = 11)	1 (9%)	10 (91%)
C in the absence of A (*n* = 41)	15 (37%)	26 (63%)
C only (*n* = 14)	5 (36%)	9 (64%)
Any case with type D (*n* = 114)	**61 (54%)**	**53 (46%)**
D in the presence of A (*n* = 9)	1 (11%)	8 (89%)
D in the absence of A (*n* = 105)	60 (57%)	45 (43%)
D only (*n* = 83)	51 (61%)	32 (39%)

Abbreviations: F/U: follow‐up; LG: Non‐invasive papillary low‐grade urothelial carcinoma; PPV: positive predictive value.

**TABLE 3:** Different cell types combination in association with outcome for the non‐surveillance cohort.

Non‐Surveillance (excluding transplantation cases) (*n* = 70 urine specimens)		
	F/U: High‐grade N (PPV)	F/U: benign/LG N (PPV)
Any case with type A (*n* = 16)	**0**	**16 (100%)**
Any case with type B (*n* = 36)	**1 (3%)**	**35 (97%)**
B in the presence of A (*n* = 10)	0	10 (100%)
B in the absence of A (*n* = 26)	1 (4%)	25 (96%)
B only (*n* = 6)	0	6 (100%)
Any case with type C (*n* = 30)	**2 (7%)**	**28 (93%)**
C in the presence of A (*n* = 9)	0	9 (100%)
C in the absence of A (*n* = 21)	2 (10%)	19 (90%)
C only (*n* = 3)	1 (33.3%)	2 (66.7%)
Any case with type D (*n* = 40)	**18 (45%)**	**22 (55%)**
D in the presence of A (*n* = 5)	0	5 (100%)
D in the absence of A (*n* = 35)	18 (51%)	17 (49%)
D only (*n* = 24)	16 (67%)	8 (33%)

Abbreviations: F/U: follow‐up; LG: Non‐invasive papillary low‐grade urothelial carcinoma; PPV: positive predictive value.

The current study includes several limitations. First, we did not record the serological results of PV infection and did not include it as an outcome to which cytology could be correlated as those were only rarely available. Similarly, we did not use the SV40 antibody to differentiate PV from PV‐like changes, as we do not use it in our routine practice. Also, we did not record the number of cells exhibiting PV and PV‐like changes in individual cases, and cannot assess whether cases with higher numbers of such cells may have a stronger association with a malignant outcome. Lastly, the heterogeneous follow‐up (histological versus clinical) may have also led to a certain degree of results contamination. This being said, the strong association of type A cells with polyoma infection/benign outcome remains a well‐established finding.

In conclusion, this is the first study to systematically analyze and report the detailed morphological features of PV‐CPE and PV‐CPE‐like cells, as well as their association with each other's, and with subsequent HGUC, using stringent morphological and clinical follow‐up criteria. Our data show that cases with cell type A morphology can be confidently diagnosed as ‘negative for HGUC, consistent with PV‐CPE’, even in the presence of associated cells with PV‐like changes (cell type B, C, or D), and regardless of the clinical context. In comparison, cases with single cells displaying a PV‐like morphology other than type A, including those with spider‐web chromatin pattern (cell B) do not always represent PV‐infected cells, and may represent degenerated HGUC cells. Whether to routinely include all such cases, or only a subset of them (especially those with cell type D) in the AUC category needs further evaluation in future study cohorts. The clinical history strongly correlated with outcome in our cohort, as patients with previous HGUC tended to show significantly higher association with a subsequent diagnosis of HGUC in contrast to the post‐transplantation patients.

## Author Contributions

Drs. Kanber, Caglar, Kassouf, Auger: Interpreting the data and drafting the manuscript; Drs Li, Brimo: Study design, collection and interpretation of data, drafting the manuscript.

## Conflicts of Interest

The authors declare no conflicts of interest.

## Data Availability

Data available on request from the authors.
